# (*Z*)-3-(3,4-Di­meth­oxy­benz­yl)-1,5-benzo­thia­zepin-4(5*H*)-one

**DOI:** 10.1107/S1600536813009598

**Published:** 2013-04-13

**Authors:** R. Selvakumar, M. Bakthadoss, S. Vijayakumar, S. Murugavel

**Affiliations:** a Department of Organic Chemistry, University of Madras, Maraimalai Campus, Chennai 600 025, India; b Department of Chemistry, Pondicherry University, Puducherry 605 014, India; c Department of Physics, Sri Balaji Chokkalingam Engineering College, Arni, Thiruvannamalai 632 317, India; d Department of Physics, Thanthai Periyar Government Institute of Technology, Vellore 632 002, India

## Abstract

In the title compound, C_18_H_17_NO_3_S, the thia­zepine ring adopts a slightly distorted twist-boat conformation. The dihedral angle between the mean plane of the benzo­thia­zepin ring system and the benzene ring is 60.3 (1)°. In the crystal, mol­ecules are linked by two pairs of inversion-related N—H⋯O and C—H⋯O hydrogen bonds, generating alternating *R*_2_^2^(8) and *R*_2_^2^(6) ring motifs, respectively, in a zigzag supra­molecular chain that runs along the *c* axis. These chains stack along the *a* axis *via* S⋯C [3.424 (2) Å] contacts. A three-dimensional supra­molecular network is consolidated by C—H⋯π and π–π inter­actions [inter-centroid distance between di­meth­oxy­benzene rings = 3.815 (1) Å]. The crystal studied was a non-merohedral twin, with a refined value of the minor twin fraction of 0.2477 (6) .

## Related literature

For background to the biology of thia­zepine derivatives and for a related structure, see: Bakthadoss *et al.* (2013 ▶). For ring-puckering parameters, see: Cremer & Pople (1975 ▶).
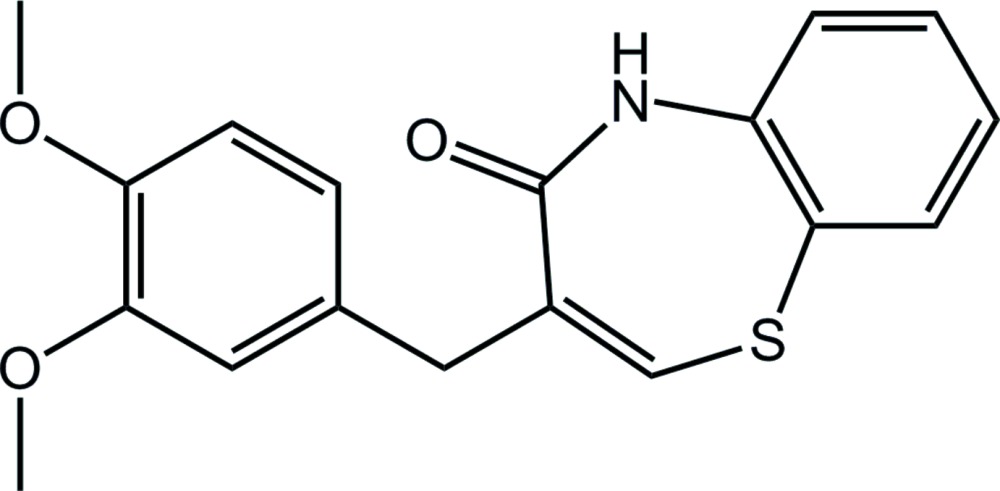


## Experimental

### 

#### Crystal data

C_18_H_17_NO_3_S
*M* *_r_* = 327.39
Orthorhombic, 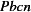

*a* = 19.966 (4) Å
*b* = 10.355 (2) Å
*c* = 15.536 (3) Å
*V* = 3212.0 (11) Å^3^
*Z* = 8
Mo *K*α radiation
μ = 0.22 mm^−1^
*T* = 293 K
0.35 × 0.20 × 0.15 mm

#### Data collection

Bruker APEXII CCD diffractometer
Absorption correction: multi-scan (*TWINABS*; Sheldrick, 1997 ▶) *T*_min_ = 0.927, *T*_max_ = 0.968
13227 measured reflections
13227 independent reflections
8413 reflections with *I* > 2σ(*I*)
*R*_int_ = 0.066

#### Refinement

*R*[*F* ^2^ > 2σ(*F* ^2^)] = 0.060
*wR*(*F* ^2^) = 0.175
*S* = 1.02
13227 reflections
211 parameters
H-atom parameters constrained
Δρ_max_ = 0.25 e Å^−3^
Δρ_min_ = −0.33 e Å^−3^

### 

Data collection: *APEX2* (Bruker, 2004 ▶); cell refinement: *APEX2* and *SAINT* (Bruker, 2004 ▶); data reduction: *SAINT* and *XPREP* (Bruker, 2004 ▶); program(s) used to solve structure: *SHELXS97* (Sheldrick, 2008 ▶); program(s) used to refine structure: *SHELXL97* (Sheldrick, 2008 ▶); molecular graphics: *ORTEP-3 for Windows* (Farrugia, 2012 ▶); software used to prepare material for publication: *SHELXL97* and *PLATON* (Spek, 2009 ▶).

## Supplementary Material

Crystal structure: contains datablock(s) global, I. DOI: 10.1107/S1600536813009598/tk5214sup1.cif

Structure factors: contains datablock(s) I. DOI: 10.1107/S1600536813009598/tk5214Isup2.hkl

Supplementary material file. DOI: 10.1107/S1600536813009598/tk5214Isup3.cml

Additional supplementary materials:  crystallographic information; 3D view; checkCIF report

## Figures and Tables

**Table 1:** Hydrogen-bond geometry (Å, °) *Cg*1 and *Cg*2 are the centroids of the C11–C16 and C2–C7 rings, respectively.

*D*—H⋯*A*	*D*—H	H⋯*A*	*D*⋯*A*	*D*—H⋯*A*
N1—H1*A*⋯O1^i^	0.86	2.02	2.863 (2)	167
C18—H18*B*⋯O3^ii^	0.96	2.53	3.445 (3)	159
C4—H4⋯*Cg*1^iii^	0.93	2.57	3.450 (2)	158
C10—H10*B*⋯*Cg*2^iv^	0.97	2.82	3.725 (2)	155

Symmetry codes: (i) 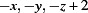; (ii) 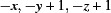; (iii) 
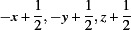
; (iv) 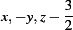.

**Fig. 1.**
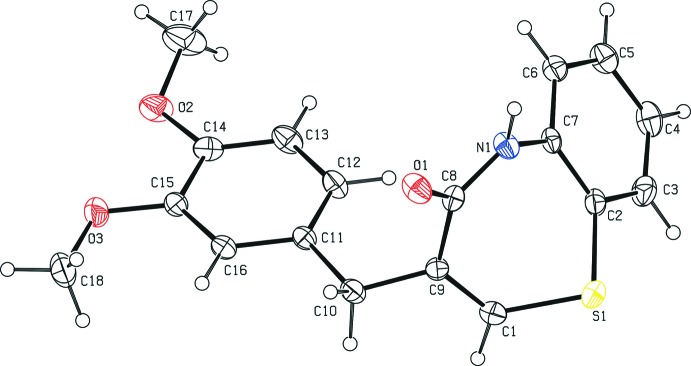
Molecular structure of the title compound showing displacement ellipsoids at the 30% probability level. H atoms are presented as a small spheres of arbitrary radii.

**Fig. 2.**
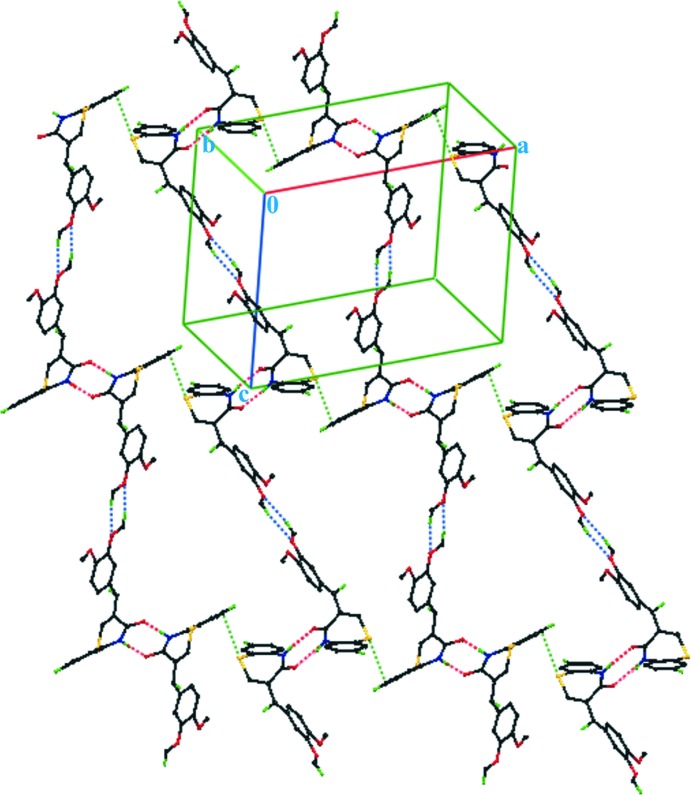
A view of the supramolecular chain showing N—H···O (red dotted lines) and C—H···O (blue dotted lines) hydrogen bonds. The chains stack along the the a axis via S···C (greeen dotted lines) short contacts.

**Fig. 3.**
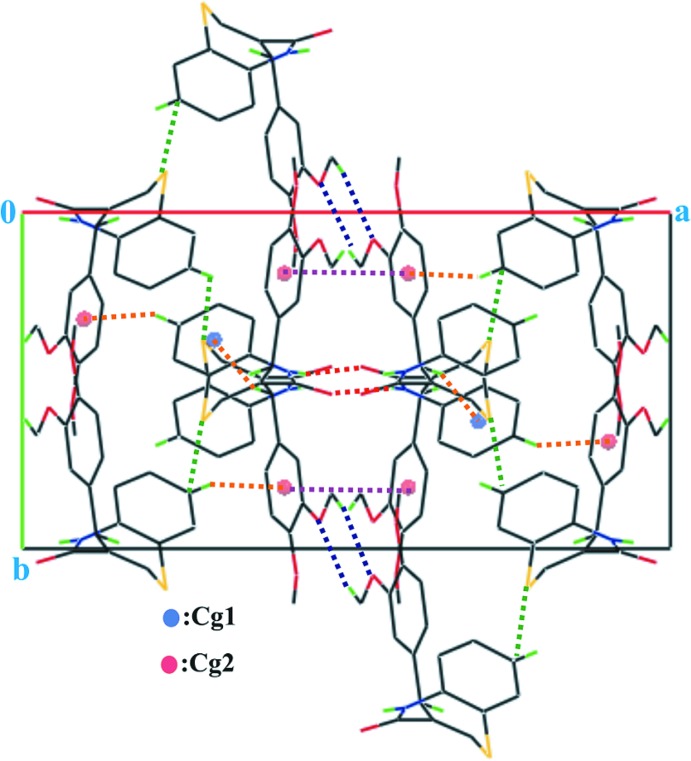
View of three-dimensional supramolecular network down the c axis. The N—H···O, C—H···O, S···C, C—H···π and π—π interactions are shown as red, blue, green, orange and purple dotted lines, respectively. Cg1 and Cg2 are the centroids of the C11–C16 and C2–C7 benzene rings, respectively.

## supplementary crystallographic information

### Comment

The background to the biology and related structure of thiazepin derivatives, has been described recently (Bakthadoss *et al.*, 2013). In view of this biological importance, the crystal structure of the title compound has been carried out and the results are presented here.

Fig. 1. shows a displacement ellipsoid plot of (I), with the atom numbering scheme. The seven membered thiazepine ring (N1/S1/C1/C2/C7/C8/C9) adopts slightly distorted twist-boat conformation as indicated by puckering parameters (Cremer & Pople, 1975): QT = 0.9884 (11) Å, φ_2_ = 359.3 (1)° and φ_3_ = 356.5 (4)°. The dihedral angle between the benzothiazepin ring system and the benzene ring is 60.3 (1)°. The atom O1 deviates by -0.895 (1) Å from the least-squares plane of the thiazepin ring. The sum of angles at N1 atom of the thiazepin ring (359.9°) is in accordance with *sp*^2^ hybridization. The geometric parameters of the title molecule agree well with those reported for a similar structure (Bakthadoss *et al.*, 2013).

In the crystal, molecules are linked by two pairs of inversion-related N1—H1A···O1 and C18—H18B···O3 hydrogen bonds, generating alternate *R_2_^2^*(8) and *R_2_^2^*(6) ring motifs, respectively, resulting in a zigzag supramolecular chain running along the *c* axis. These chains stack along the *a* axis by S1···C4^v^ = 3.424 (2) Å (Symmetry code:(v) = 1/2 - *x*, -1/2 + *y*, *z*) short contacts (Fig. 2 and Table 1). A three-dimensional supramolecular network is consolidated by C4—H4···*Cg*1^iii^ (symmetry code: (iii) = 1/2 - *x*, 1/2 - *y*, 1/2 + *z*) and C10—H10B···*Cg*2^iv^ (symmetry code: (iv) = *x*, -*y*, -1/2 + *z*) hydrogen bonds and *Cg*1—*Cg*1^vi^ = 3.815 (1) Å (symmetry code: (vi) = -*x*, *y*, 3/2 - *z*) interactions (Fig. 3 and Table 1; *Cg*1 and *Cg*2 are the centroids of the C11–C16 and C2–C7 benzene rings, respectively).

### Experimental

A mixture of (*Z*)-methyl 2-(bromomethyl)-3-(3,4-dimethoxyphenyl)acrylate 2 mmol) and *o*-aminothiophenol (2 mmol) in the presence of potassium *tert*-butoxide (4.8 mmol) in dry THF (10 ml) was stirred at room temperature for 1 h. After the completion of the reaction as indicated by TLC, the reaction mixture was concentrated and the resulting crude mass was diluted with water (20 ml) and extracted with ethyl acetate (3 *x* 20 ml). The organic layer was washed with brine (2 *x* 20 ml) and dried over anhydrous sodium sulfate. The organic layer was concentrated, which successfully provide the crude final product ((*Z*)-3-(3,4-dimethoxybenzyl)benzo[*b*][1,4]thiazepin-4(5*H*)-one). This product was purified by column chromatography on silica gel to afford the title compound in good yield (44%). Single crystals suitable for X-ray diffraction were obtained by slow evaporation of its ethylacetate solution at room temperature.

### Refinement

The investigated crystal was found to be a two-component rotational twin. The data for both components were integrated using *SAINT* and scaled with *TWINABS*. Final refinement was perfomed using a *HKLF5* file generated by *TWINABS* with a *BASF* parameter (0.2477 (6)). Owing to poor agreement, the reflections (-7 5 5), (-7 5 7), (-8 4 - 5), (8 5 5), (2 0 0), (-8 - 5 5), (8 5 - 5), (8 - 5 5), (8 - 5 -5), (-8 - 5 -5), (-8 - 6 5), (-7 5 3), (-8 - 6 -5), (7 - 5 -3), (14 2 2) and (8 - 6 5) were omitted from the final cycles of refinement. All the H atoms were positioned geometrically and constrained to ride on their parent atom with C—H = 0.93–0.97 Å and N—H = 0.86 Å, and with *U*_iso_(H)=1.5*U*_eq_ for methyl H atoms and 1.2*U*_eq_(C) for other H atoms.

### Crystal data

**Table:**

C_18_H_17_NO_3_S	*F*(000) = 1376
*M**_r_* = 327.39	*D*_x_ = 1.354 Mg m^−^^3^
Orthorhombic, *P**b**c**n*	Mo *K*α radiation, λ = 0.71073 Å
Hall symbol: -P 2n 2ab	Cell parameters from 2790 reflections
*a* = 19.966 (4) Å	θ = 2.2–24.9°
*b* = 10.355 (2) Å	µ = 0.22 mm^−^^1^
*c* = 15.536 (3) Å	*T* = 293 K
*V* = 3212.0 (11) Å^3^	Block, colourless
*Z* = 8	0.35 × 0.20 × 0.15 mm

### Data collection

**Table:**

Bruker APEXII CCD diffractometer	13227 independent reflections
Radiation source: fine-focus sealed tube	8413 reflections with *I* > 2σ(*I*)
Graphite monochromator	*R*_int_ = 0.066
Detector resolution: 10.0 pixels mm^-1^	θ_max_ = 24.9°, θ_min_ = 2.2°
ω scans	*h* = −23→23
Absorption correction: multi-scan (TWINABS; Sheldrick, 1997)	*k* = −12→9
*T*_min_ = 0.927, *T*_max_ = 0.968	*l* = −18→18
13227 measured reflections	

### Refinement

**Table:**

Refinement on *F*^2^	Primary atom site location: structure-invariant direct methods
Least-squares matrix: full	Secondary atom site location: difference Fourier map
*R*[*F*^2^ > 2σ(*F*^2^)] = 0.060	Hydrogen site location: inferred from neighbouring sites
*wR*(*F*^2^) = 0.175	H-atom parameters constrained
*S* = 1.02	*w* = 1/[σ^2^(*F*_o_^2^) + (0.0582*P*)^2^] where *P* = (*F*_o_^2^ + 2*F*_c_^2^)/3
13227 reflections	(Δ/σ)_max_ = 0.001
211 parameters	Δρ_max_ = 0.25 e Å^−^^3^
0 restraints	Δρ_min_ = −0.33 e Å^−^^3^

### Special details

**Table:**

Geometry. All e.s.d.'s (except the e.s.d. in the dihedral angle between two l.s. planes) are estimated using the full covariance matrix. The cell e.s.d.'s are taken into account individually in the estimation of e.s.d.'s in distances, angles and torsion angles; correlations between e.s.d.'s in cell parameters are only used when they are defined by crystal symmetry. An approximate (isotropic) treatment of cell e.s.d.'s is used for estimating e.s.d.'s involving l.s. planes.
Refinement. Refinement of *F*^2^ against ALL reflections. The weighted *R*-factor *wR* and goodness of fit *S* are based on *F*^2^, conventional *R*-factors *R* are based on *F*, with *F* set to zero for negative *F*^2^. The threshold expression of *F*^2^ > σ(*F*^2^) is used only for calculating *R*-factors(gt) *etc*. and is not relevant to the choice of reflections for refinement. *R*-factors based on *F*^2^ are statistically about twice as large as those based on *F*, and *R*- factors based on ALL data will be even larger.

### Fractional atomic coordinates and isotropic or equivalent isotropic displacement parameters (Å^2^)

**Table:**

	*x*	*y*	*z*	*U*_iso_*/*U*_eq_	
C2	0.21158 (10)	0.01584 (18)	1.02679 (11)	0.0320 (5)	
C3	0.26554 (10)	0.0619 (2)	1.07376 (13)	0.0401 (5)	
H3	0.3072	0.0225	1.0683	0.048*	
C4	0.25791 (10)	0.1650 (2)	1.12822 (13)	0.0434 (6)	
H4	0.2939	0.1935	1.1611	0.052*	
C5	0.19688 (11)	0.2264 (2)	1.13416 (12)	0.0405 (5)	
H5	0.1921	0.2978	1.1700	0.049*	
C6	0.14302 (10)	0.18270 (19)	1.08740 (11)	0.0339 (5)	
H6	0.1020	0.2249	1.0913	0.041*	
C7	0.14978 (9)	0.07580 (18)	1.03449 (10)	0.0265 (4)	
C8	0.07914 (10)	−0.00895 (17)	0.91369 (11)	0.0300 (5)	
C9	0.13353 (9)	−0.00907 (17)	0.84820 (11)	0.0277 (5)	
C1	0.19328 (11)	−0.05969 (19)	0.86307 (11)	0.0359 (5)	
H1	0.2230	−0.0622	0.8170	0.043*	
C10	0.11450 (10)	0.04089 (18)	0.76116 (11)	0.0332 (5)	
H10A	0.0735	−0.0016	0.7432	0.040*	
H10B	0.1493	0.0170	0.7206	0.040*	
C11	0.10408 (8)	0.18544 (17)	0.75624 (10)	0.0267 (4)	
C12	0.12408 (10)	0.2698 (2)	0.81859 (11)	0.0369 (5)	
H12	0.1440	0.2384	0.8685	0.044*	
C13	0.11523 (10)	0.4015 (2)	0.80865 (12)	0.0406 (5)	
H13	0.1286	0.4574	0.8522	0.049*	
C14	0.08704 (10)	0.45013 (18)	0.73534 (12)	0.0345 (5)	
C15	0.06728 (9)	0.36546 (19)	0.67025 (11)	0.0305 (5)	
C16	0.07503 (9)	0.23488 (19)	0.68158 (10)	0.0298 (5)	
H16	0.0607	0.1785	0.6388	0.036*	
C18	0.02274 (13)	0.3361 (2)	0.52964 (13)	0.0661 (8)	
H18A	0.0616	0.2902	0.5099	0.099*	
H18B	0.0042	0.3852	0.4830	0.099*	
H18C	−0.0100	0.2756	0.5502	0.099*	
C17	0.08134 (13)	0.6638 (2)	0.78994 (14)	0.0715 (8)	
H17A	0.0536	0.6325	0.8359	0.107*	
H17B	0.0665	0.7483	0.7730	0.107*	
H17C	0.1270	0.6684	0.8091	0.107*	
N1	0.09093 (8)	0.02648 (15)	0.99504 (9)	0.0325 (4)	
H1A	0.0573	0.0180	1.0291	0.039*	
O1	0.02201 (7)	−0.04165 (14)	0.89186 (8)	0.0423 (4)	
O3	0.04118 (7)	0.42072 (13)	0.59744 (8)	0.0462 (4)	
O2	0.07680 (8)	0.57865 (13)	0.71876 (9)	0.0498 (4)	
S1	0.22192 (3)	−0.12256 (6)	0.96130 (3)	0.04692 (18)	

### Atomic displacement parameters (Å^2^)

**Table:**

	*U*^11^	*U*^22^	*U*^33^	*U*^12^	*U*^13^	*U*^23^
C2	0.0340 (13)	0.0353 (12)	0.0267 (10)	0.0018 (10)	−0.0032 (9)	0.0069 (9)
C3	0.0285 (13)	0.0482 (15)	0.0437 (12)	0.0032 (10)	−0.0051 (9)	0.0068 (11)
C4	0.0369 (15)	0.0530 (15)	0.0404 (12)	−0.0140 (12)	−0.0120 (10)	0.0064 (11)
C5	0.0447 (14)	0.0402 (13)	0.0366 (11)	−0.0090 (11)	−0.0044 (10)	−0.0018 (10)
C6	0.0347 (13)	0.0359 (13)	0.0311 (10)	−0.0005 (10)	0.0000 (9)	0.0014 (9)
C7	0.0256 (12)	0.0326 (12)	0.0214 (9)	−0.0028 (9)	−0.0011 (8)	0.0067 (9)
C8	0.0302 (13)	0.0278 (12)	0.0320 (10)	0.0017 (9)	−0.0025 (9)	0.0044 (9)
C9	0.0310 (12)	0.0249 (11)	0.0271 (9)	0.0008 (9)	−0.0016 (8)	−0.0015 (8)
C1	0.0391 (14)	0.0386 (13)	0.0300 (10)	0.0034 (10)	0.0048 (9)	−0.0031 (9)
C10	0.0394 (13)	0.0351 (12)	0.0252 (9)	−0.0004 (9)	0.0006 (8)	−0.0022 (9)
C11	0.0263 (12)	0.0297 (11)	0.0242 (9)	−0.0046 (9)	0.0029 (8)	0.0008 (9)
C12	0.0463 (14)	0.0395 (13)	0.0248 (10)	−0.0052 (11)	−0.0030 (9)	−0.0008 (9)
C13	0.0524 (15)	0.0357 (14)	0.0336 (11)	−0.0129 (11)	0.0002 (10)	−0.0083 (10)
C14	0.0406 (14)	0.0246 (12)	0.0384 (11)	−0.0057 (9)	0.0126 (10)	−0.0006 (10)
C15	0.0324 (13)	0.0324 (12)	0.0265 (10)	0.0009 (9)	0.0052 (8)	0.0046 (9)
C16	0.0319 (13)	0.0310 (12)	0.0263 (10)	−0.0039 (9)	0.0006 (8)	−0.0034 (9)
C18	0.111 (2)	0.0510 (16)	0.0358 (13)	0.0186 (15)	−0.0196 (13)	−0.0014 (11)
C17	0.118 (2)	0.0342 (15)	0.0621 (16)	−0.0121 (15)	0.0208 (15)	−0.0165 (13)
N1	0.0229 (10)	0.0474 (11)	0.0273 (8)	−0.0062 (8)	0.0031 (7)	0.0024 (8)
O1	0.0294 (9)	0.0628 (10)	0.0346 (7)	−0.0108 (7)	−0.0031 (6)	−0.0053 (7)
O3	0.0675 (11)	0.0373 (9)	0.0338 (8)	0.0099 (7)	−0.0051 (7)	0.0047 (7)
O2	0.0797 (12)	0.0244 (8)	0.0452 (8)	−0.0014 (8)	0.0113 (8)	−0.0036 (7)
S1	0.0543 (4)	0.0454 (4)	0.0411 (3)	0.0223 (3)	−0.0086 (3)	0.0000 (3)

### Geometric parameters (Å, º)

**Table:**

C2—C3	1.386 (3)	C10—H10B	0.9700
C2—C7	1.386 (2)	C11—C12	1.364 (2)
C2—S1	1.770 (2)	C11—C16	1.394 (2)
C3—C4	1.371 (3)	C12—C13	1.383 (3)
C3—H3	0.9300	C12—H12	0.9300
C4—C5	1.377 (3)	C13—C14	1.367 (3)
C4—H4	0.9300	C13—H13	0.9300
C5—C6	1.374 (3)	C14—O2	1.371 (2)
C5—H5	0.9300	C14—C15	1.395 (3)
C6—C7	1.385 (3)	C15—O3	1.370 (2)
C6—H6	0.9300	C15—C16	1.372 (3)
C7—N1	1.420 (2)	C16—H16	0.9300
C8—O1	1.237 (2)	C18—O3	1.419 (2)
C8—N1	1.337 (2)	C18—H18A	0.9600
C8—C9	1.488 (3)	C18—H18B	0.9600
C9—C1	1.323 (3)	C18—H18C	0.9600
C9—C10	1.497 (2)	C17—O2	1.417 (2)
C1—S1	1.7549 (19)	C17—H17A	0.9600
C1—H1	0.9300	C17—H17B	0.9600
C10—C11	1.513 (2)	C17—H17C	0.9600
C10—H10A	0.9700	N1—H1A	0.8600
			
C3—C2—C7	119.50 (18)	C16—C11—C10	117.55 (16)
C3—C2—S1	119.38 (16)	C11—C12—C13	120.97 (18)
C7—C2—S1	121.07 (14)	C11—C12—H12	119.5
C4—C3—C2	120.44 (19)	C13—C12—H12	119.5
C4—C3—H3	119.8	C14—C13—C12	120.58 (18)
C2—C3—H3	119.8	C14—C13—H13	119.7
C3—C4—C5	119.93 (19)	C12—C13—H13	119.7
C3—C4—H4	120.0	C13—C14—O2	125.16 (17)
C5—C4—H4	120.0	C13—C14—C15	119.27 (18)
C6—C5—C4	120.3 (2)	O2—C14—C15	115.57 (17)
C6—C5—H5	119.8	O3—C15—C16	124.07 (17)
C4—C5—H5	119.8	O3—C15—C14	116.31 (17)
C5—C6—C7	120.03 (19)	C16—C15—C14	119.61 (17)
C5—C6—H6	120.0	C15—C16—C11	121.03 (17)
C7—C6—H6	120.0	C15—C16—H16	119.5
C6—C7—C2	119.72 (17)	C11—C16—H16	119.5
C6—C7—N1	117.57 (17)	O3—C18—H18A	109.5
C2—C7—N1	122.54 (17)	O3—C18—H18B	109.5
O1—C8—N1	119.77 (17)	H18A—C18—H18B	109.5
O1—C8—C9	119.02 (17)	O3—C18—H18C	109.5
N1—C8—C9	121.21 (18)	H18A—C18—H18C	109.5
C1—C9—C8	122.60 (17)	H18B—C18—H18C	109.5
C1—C9—C10	121.56 (17)	O2—C17—H17A	109.5
C8—C9—C10	115.60 (16)	O2—C17—H17B	109.5
C9—C1—S1	126.33 (15)	H17A—C17—H17B	109.5
C9—C1—H1	116.8	O2—C17—H17C	109.5
S1—C1—H1	116.8	H17A—C17—H17C	109.5
C9—C10—C11	114.99 (15)	H17B—C17—H17C	109.5
C9—C10—H10A	108.5	C8—N1—C7	130.72 (16)
C11—C10—H10A	108.5	C8—N1—H1A	114.6
C9—C10—H10B	108.5	C7—N1—H1A	114.6
C11—C10—H10B	108.5	C15—O3—C18	116.96 (16)
H10A—C10—H10B	107.5	C14—O2—C17	116.60 (16)
C12—C11—C16	118.50 (17)	C1—S1—C2	99.30 (9)
C12—C11—C10	123.88 (16)		
			
C7—C2—C3—C4	0.7 (3)	C11—C12—C13—C14	−1.0 (3)
S1—C2—C3—C4	−176.75 (15)	C12—C13—C14—O2	−179.20 (17)
C2—C3—C4—C5	−2.3 (3)	C12—C13—C14—C15	−0.1 (3)
C3—C4—C5—C6	1.7 (3)	C13—C14—C15—O3	−178.15 (17)
C4—C5—C6—C7	0.5 (3)	O2—C14—C15—O3	1.0 (2)
C5—C6—C7—C2	−2.1 (3)	C13—C14—C15—C16	1.5 (3)
C5—C6—C7—N1	173.30 (16)	O2—C14—C15—C16	−179.36 (16)
C3—C2—C7—C6	1.5 (3)	O3—C15—C16—C11	177.80 (17)
S1—C2—C7—C6	178.92 (13)	C14—C15—C16—C11	−1.8 (3)
C3—C2—C7—N1	−173.68 (16)	C12—C11—C16—C15	0.7 (3)
S1—C2—C7—N1	3.7 (2)	C10—C11—C16—C15	−176.41 (16)
O1—C8—C9—C1	−133.7 (2)	O1—C8—N1—C7	−175.13 (18)
N1—C8—C9—C1	46.7 (3)	C9—C8—N1—C7	4.5 (3)
O1—C8—C9—C10	40.8 (2)	C6—C7—N1—C8	133.3 (2)
N1—C8—C9—C10	−138.76 (18)	C2—C7—N1—C8	−51.4 (3)
C8—C9—C1—S1	−5.3 (3)	C16—C15—O3—C18	−1.4 (3)
C10—C9—C1—S1	−179.56 (14)	C14—C15—O3—C18	178.15 (18)
C1—C9—C10—C11	−113.1 (2)	C13—C14—O2—C17	−15.3 (3)
C8—C9—C10—C11	72.2 (2)	C15—C14—O2—C17	165.61 (18)
C9—C10—C11—C12	14.1 (3)	C9—C1—S1—C2	−58.0 (2)
C9—C10—C11—C16	−168.94 (16)	C3—C2—S1—C1	−125.91 (16)
C16—C11—C12—C13	0.7 (3)	C7—C2—S1—C1	56.68 (17)
C10—C11—C12—C13	177.59 (18)		

### Hydrogen-bond geometry (Å, º)

Cg1 and Cg2 are the centroids of the C11–C16 and C2–C7 rings, respectively.

**Table:**

*D*—H···*A*	*D*—H	H···*A*	*D*···*A*	*D*—H···*A*
N1—H1*A*···O1^i^	0.86	2.02	2.863 (2)	167
C18—H18*B*···O3^ii^	0.96	2.53	3.445 (3)	159
C4—H4···*Cg*1^iii^	0.93	2.57	3.450 (2)	158
C10—H10*B*···*Cg*2^iv^	0.97	2.82	3.725 (2)	155

Symmetry codes: (i) −*x*, −*y*, −*z*+2; (ii) −*x*, −*y*+1, −*z*+1; (iii) −*x*+1/2, −*y*+1/2, *z*+1/2; (iv) *x*, −*y*, *z*−3/2.
